# Enhanced hypertension care through private clinics in Pakistan: a cluster randomised trial

**DOI:** 10.3399/bjgpopen18X101617

**Published:** 2019-01-23

**Authors:** Muhammad Amir Khan, Nida Khan, John D Walley, Shaheer Ellahi Khan, Joseph Hicks, Faisal Imtiaz Sheikh, Muhammad Ahmar Khan, Muhammad Ali, Maqsood Ahmed, Haroon Jehangir Khan, Rony Zachariah

**Affiliations:** 1 Chief Coordinating Professional, Association for Social Development, Islamabad, Pakistan; 2 Project Coordinator, Association for Social Development, Islamabad, Pakistan; 3 Professor of International Public Health, Nuffield Centre for International Health and Development, Leeds Institute of Health Sciences, University of Leeds, Leeds, UK; 4 Assistant Professor, Humanities and Social Sciences Department, Bahria University, Islamabad, Pakistan; 5 Senior Medical Statistician, Nuffield Centre for International Health and Development, Leeds Institute of Health Sciences, Leeds, UK; 6 Research Coordinator, Association for Social Development, Islamabad, Pakistan; 7 Research Coordinator, Association for Social Development, Islamabad, Pakistan; 8 Research Assistant, Association for Social Development, Islamabad, Pakistan; 9 Senior Professional, Association for Social Development, Islamabad, Pakistan; 10 Director, NCD & Mental Health, Directorate General of Health Services, Lahore, Pakistan; 11 General Coordinator and Strategic Advisor, Operational Research, Médecins Sans Frontières, Brussels, Belgium

**Keywords:** Cluster randomised controlled trial, primary private clinics, hypertension, contextualised care package, primary care, general practice

## Abstract

**Background:**

Hypertension in Pakistan affects 33% of people aged ≥45 years, and in urban areas around 70% of basic health care occurs in private facilities.

**Aim:**

To assess whether enhanced care at urban private clinics resulted in better control of hypertension, cardiovascular disease (CVD) risk factors, and treatment adherence.

**Design & setting:**

A two-arm cluster randomised controlled trial was conducted at 26 private clinics (in three districts of Punjab) between January 2015–September 2016. Both arms had enhanced screening and diagnosis of hypertension and related conditions, and patient recording processes. Intervention facilities also had a clinical care guide, additional drugs for hypertension, a patient lifestyle education flipchart, associated training, and mobile phone follow-up.

**Method:**

Clinics were randomised in a 1:1 ratio (sealed envelope lottery method). A total of 574 intervention and 564 control patients in 13 clusters in each arm were recruited (male and female, aged ≥25 years, systolic blood pressure [SBP] >140 mmHg, and/or diastolic blood pressure [DBP] >90 mmHg). The primary outcome was change in SBP from baseline to 9-month follow-up.

Staff and patients were not blinded, but outcome assessors were blinded.

**Results:**

Nine-month primary outcomes were available for 522/574 (90.9%) intervention and 484/564 (85.8%) control participants (all clusters). The unadjusted cluster-level analysis results were as follows: mean intervention outcome was -25.2 mmHg (95% confidence intervals [CI] = -29.9 to

-20.6); mean control outcome was -9.4 mmHg (95% CI = 21.2 to 2.2); and mean control–intervention difference was 15.8 (95% CI = 3.6 to 28.0; *P* = 0.01).

**Conclusion:**

The findings and separate process evaluation support the scaling of an integrated CVD–hypertension care intervention in urban private clinics in areas lacking public primary care in Pakistan.

## How this fits in

Engagement of private clinics in delivering an integrated care for non-communicable diseases, including hypertension, is a programme priority.^1^ The key care considerations for hypertension and other CVD risk factors are long-term care and lifestyle change; for example, healthy eating, activity, and tobacco use cessation. The effectiveness of delivering integrated hypertension-CVD care components at private clinics has never been evaluated before in Pakistan. Evidence was required for the programme to take informed intervention scaling decisions.

## Introduction

Hypertension and related CVDs cause high mortality globally.^2^ The level of detection, treatment, and control of hypertension is low, especially in resource-limited countries, leading to complications such as stroke, and heart and renal failure.^3–5^ Globally, 54% of strokes and 47% of ischaemic heart disease can be attributed to high blood pressure (SBP >140 mmHg and/or DBP >90 mmHg).^6^

In Pakistan, non-communicable diseases (NCDs) account for 42% of deaths annually.^7^ Hypertension is common, affecting 18% of adults aged >15 years and 33% of those >45 years.^8,9^ A hospital-based study of adult patients with hypertension in Pakistan also showed that 36% had hyperlipidaemia.^10^ Compared with those with hypertension alone, the risk of developing CVD is two to three times higher in those who have hypertension with diabetes and hyperlipidaemia.^11^ Treatment adherence and lifestyle modifications to diet, daily activity, and smoking cessation are known to be important in hypertension care.^12,13^ Effective management of hypertension has been a challenge in Pakistan. Around two-thirds of the adult population do not get their BP checked,^14^ and, of those diagnosed, only 34% receive appropriate treatment. Furthermore, only 3% of patients with hypertension achieve the BP control target of 140/90 mmHg.^9^ The provincial department of health has recently established a programme to coordinate efforts for an enhanced management of NCDs (including hypertension–CVD, diabetes, asthma/chronic obstructive pulmonary disease, and mental health) at public and private healthcare facilities in the Punjab province.

In Pakistan, private health clinics account for 70% of basic health care in the country.^15^ The majority of the private clinics are small entities, run by one or more doctors and allied staff. In general, the care at private clinics does not follow internationally agreed standards of diagnosis and treatment. Furthermore, the care arrangements at private clinics are not designed to offer long-term care of chronic conditions. However, there is potential to enable and engage these private entities in delivering programme-endorsed NCD care to those currently not able or willing to access public service.^15,16^

In Pakistan, weak regulatory function makes public–private partnership a possible way to enable and engage the private clinics in delivering better health care. The non-franchise variants of the public–private partnership model have been developed and tested for tuberculosis (TB) care in low-resource settings of more than one south Asian country.^17^ In Pakistan, the district-steered public–private partnership model has also been adapted and used for delivering malaria care in poor urban settings.^18^ In Pakistan, the TB programme-endorsed care through a non-franchise partnership model has reported sharing around 20% of TB and/or malaria care load, with ≥90% treatment success, in the respective districts.^18^

There are two considerations that make the hypertension care different from that for TB and malaria: life-long care to control (rather than cure) the condition; and both drug treatment and lifestyle modification to achieve disease control. The delivery of integrated hypertension (chronic disease) care at private clinics has not been evaluated before in Pakistan; there is, therefore, a lack of evidence for the programme and its partners to inform possible scaling.

This district-steered partnership model was adapted for engaging the private clinics to deliver enhanced care for hypertension and associated conditions. The enhanced care included: (a) a standardised approach to diagnosis and drug treatment; (b) education about lifestyle change (that is, diet, exercise, and smoking cessation); and (c) active follow-up of patients to improve retention in care. The study aimed to assess whether the enhanced care at private clinics resulted in better control of BP and associated conditions, for example, hyperlipidaemia.

## Method

### Study design, setting, and participants

The study protocol has been published previously.^19^ A two-arm cluster randomised controlled intervention trial was conducted at 26 selected private clinics (clusters), in three districts of Punjab province (Sargodha, Mandi-Bahauddin, and Kasur), during the period January 2015–September 2016. A cluster design was used primarily because the intervention was delivered by doctors who required training and resources to deliver the intervention, and it would not have been possible for doctors to effectively treat patients under usual care and/or control conditions once trained on the intervention processes and provided with the intervention resources.

In the three districts, 66 private clinics that had been already mapped, selected, and engaged (for ≥1 years) in the delivery of TB care were listed. All the listed clinics, when selected, were found qualified on the minimal eligibility criteria, that is ≥1 room with furniture and/or equipment, one doctor, and one clinic assistant, daily outpatient load ≥20, and willingness to participate in the partnership arrangement. Then 26 clinics were randomly selected, proportionately, from each district. The research staff informed each clinic in-charge (doctor) and asked for their consent to participate in the trial. After consent, the 26 clinics were randomised into intervention and control arms (1:1 basis). A designated doctor and allied staff at each of the 26 clinics were trained on standardised hypertension diagnosis and record-keeping; whereas at 13 intervention clinics, the designated doctor was trained on the standardised clinical care, and allied staff members were trained on lifestyle education and patient follow-up.

Patients aged ≥25 years were included in the trial after their eligibility was assessed and their (informed) consent to participate in the trial was obtained. Those excluded were pregnant patients, those with already known hypertension or secondary hypertension, those with advanced chronic disease, those on CVD treatment previously, or those who knew they would not be residing in the catchment area for the required follow-up period of 9 months. Every eligible patient attending the clinic, and consenting to participate, was enrolled until the required number was achieved in all clusters.

### Randomisation and blinding

The 26 clinics in three districts (that is, *n* = 8, *n* =8, and *n* =10) were randomised to the intervention and control arm on a 1:1 basis using a lottery method. The number of clusters from each district were chosen proportionately, but randomisation was not stratified by district. From 26 sealed envelopes, each containing a clinic name, in the presence of trial steering committee members, a staff member of the provincial directorate randomly selected 13 envelopes for the intervention arm and 13 for the control arm. To avoid protocol contamination, the staff at the intervention and control clinics were trained separately. It was not possible to blind the healthcare staff and patients to the allocation of trial clinics owing to the nature of intervention. However, outcome assessors were blinded to clinic allocation and any previous clinical measurements for patients.

### Package of care at intervention and control clinics

The doctors and allied staff in both cluster arms were given the same training on initial patient screening (for example, overweight), diagnosing hypertension, and using a chronic disease card for recording. For study purposes, both arms were provided with resources over and above what was available in usual care. These included provision of disease cards (to record data at baseline and follow-up), electronic sphygmomanometer, weighing scale, glucometer and glucose test strips, thiazide tablets, and vouchers for free testing of serum cholesterol at identified laboratories.

In the intervention arm, along with these resources, the enhanced management of hypertension and associated conditions was supported by providing: (a) a context-adapted desk guide for step-by-step clinical care, including adding drugs and amending dosage of drugs for hypertension and associated conditions; (b) contextualised pictorial tool to educate patients regarding hypertension care and lifestyle change; (c) training of doctors and 'paramedics' (qualified technical staff members with training in general medical care provision) to deliver care using the desk guide and education materials; and (d) mobile phone and pre-paid cards for the allied staff to message and/or call patients about follow-up visits.

Each registered patient was asked to visit the clinic on a monthly basis, or earlier if required for any medical reason. At each monthly follow-up visit, the patients were clinically assessed, prescribed, dispensed (only thiazide), educated, and their records were updated. At intervention clinics, all these care tasks were carried out as per recommended intervention protocols; whereas at control clinics, the care was delivered as per routine practice at the respective clinic. At intervention clinics, patient adherence to follow-up visits was also supported through mobile phone reminders as and when needed. To keep the trial evidence relevant to the real-life programme implementation processes, any special measure for an enhanced staff adherence to the intervention protocol was deliberately avoided. Staff and patient adherence, and challenges to the intervention protocols, have been studied and published in this journal,^20^ and the guides and tools are available at http://comdis-hsd.leeds.ac.uk/resources/ncd-care-package/.

### Data collection and management

The data were collected, as a part of care delivery process, on dedicated NCD record cards. In both arms, recruited patients’ baseline clinical data on examination (for example, BP, blood glucose, serum cholesterol, and HbA1c) and prescription (that is drugs and dosage) were recorded by the doctor, and non-clinical data (for example, age, contact details, height, and weight) were recorded by the allied staff. Patients were followed-up for an outcome measurement at 9 months of treatment. Those who were deceased or did not turn up for the 9-month follow-up for 3 months were declared as lost to follow-up.

Patient NCD card data were stored on an SPSS database (version 17) by trained data entry staff with regular quality checks by a supervisor.^21^

### Outcome measures

All outcomes were measured at the individual-level. The primary treatment outcome was the mean change in SBP (mmHg) between baseline and 9 months. The secondary outcomes were: (a) mean change in DBP (mmHg), total serum cholesterol (mg/dL), and HbA1c (%); (b) 9-month control of SBP (≤140 mmHg), serum cholesterol (<200 mg/dL), and HbA1c (≤7%); (c) reported rate of smoking cessation among smokers; and (d) reported cardiovascular and cerebrovascular events.

### Sample size

In the protocol sample size, it was initially planned for a fixed, recruitable total of 12 clusters, with 76 patients per cluster. However, in response to a slower than expected patient recruitment rate per cluster in the piloting phase, the number of clusters was increased to achieve the desired number of patients recruited. Therefore, the ultimate aim was to recruit 26 clusters (13 per arm), assuming an average of 35 patients would be recruited in each cluster with a 25% loss to follow-up (that is, 26 patients followed-up per cluster), giving a total of 912 patients recruited with 676 followed-up. Based on the original protocol assumptions of an outcome standard deviation of 11^22^ and an intra-cluster correlation coefficient of 0.02,^23^ this gave 80% power (for a 5% level of significance and two-tailed test) to detect a difference in the mean change in SBP of ≥2.1 mmHg between the intervention and the control arm.^24,25^

### Statistical analysis

The methods used were those that were suitable for cluster randomised controlled trials having a relatively small number of clusters per arm.^26^ To analyse the crude effect of the intervention on the primary outcome, cluster-level summary values were calculated, which were based on the mean outcome in each cluster. An independent *t*-test was then used to calculate the treatment effect as the mean difference in the primary outcome between treatment arms (control minus intervention), along with the associated *P* value and 95% CIs. A two-stage approach was then used to adjust for potentially confounding covariates. First, a linear regression model was fitted to the individual-level primary outcome data, which adjusted for the covariates of interest excluding the treatment effect. The covariates were chosen before analysis and included sex, years of education, age, SBP at baseline, serum cholesterol at baseline, HbA1c at baseline, and BMI at baseline. This model was then used to calculate a covariate-adjusted difference-residual for each cluster, by calculating the mean difference between the observed and model-predicted outcome for every cluster. These cluster-level difference residuals were then analysed using an independent *t*-test as described above to estimate the covariate-adjusted intervention effect.

The continuous secondary outcomes were analysed using the same methods as the primary outcome analysis. For the binary secondary outcomes, the cluster-level proportions were calculated first, and the crude difference in outcome proportions were estimated between treatment arms (control minus intervention; that is, the risk difference), along with the associated *P *values and 95% confidence intervals, using an independent *t*-test. To adjust for covariates, the same two-stage process as described was used, but instead using a logistic regression model for the individual-level outcome data to calculate cluster-level difference-residuals, which were then analysed using an independent *t*-test to estimate the covariate-adjusted intervention effect.^26^

The crude and covariate-adjusted extent of effect modification due to sex on the primary outcome was estimated by calculating, for each cluster, the mean female-minus-male difference in the primary outcome (for the covariate-adjusted analysis, cluster-level difference residuals were used in place of raw means, calculated as explained above, but without controlling for sex). These crude and covariate-adjusted male-female difference values were then compared between treatment arms (control versus intervention) using an independent *t*-test to estimate the crude and covariate-adjusted between sex difference in the treatment effect.

Statistical significance was set at 5% and two-sided *P* values calculated. All data were analysed as per the original allocation of clusters, and when patients were missing outcome and/or covariate data, they were excluded from all relevant analyses (complete case analysis).

## Results

**Figure 1.**
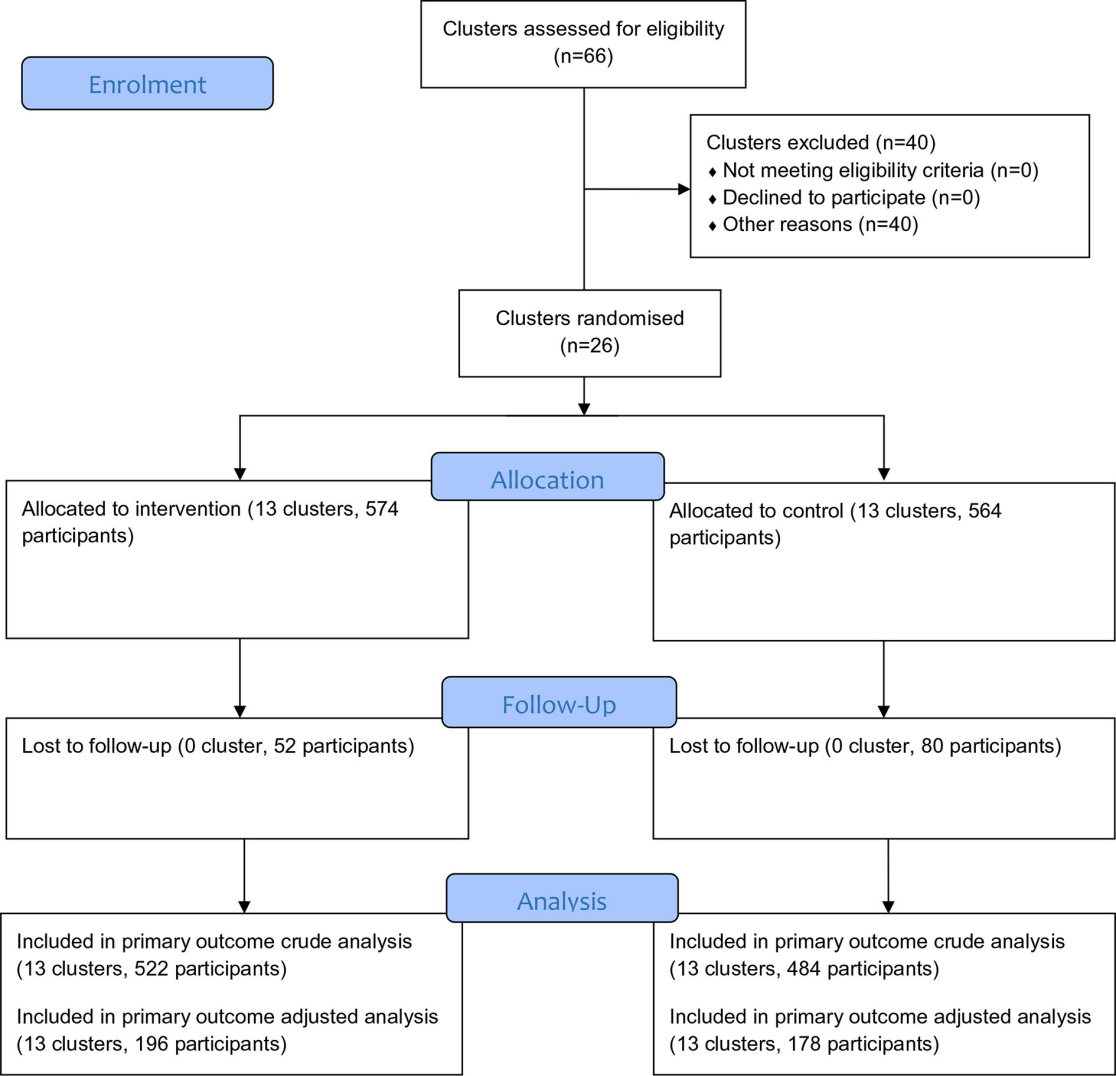
CONSORT trial flow chart

Thirteen private health facilities were randomised to each arm in October 2014, and recruited a total of 1138 patients between 28 November 2014–15 December 2015, completing all follow-ups by 16 September 2016. Trial flow, including loss to follow-up, is shown in Figure 1. Baseline characteristics of individuals in both arms are shown in Table 1, and appeared well balanced. There were no substantive differences between the crude and covariate-adjusted outcome results, although the covariate-adjusted results were based on small sample sizes due to missing covariate data.

For all crude analyses, except for the change in HbA1c and glycaemic control, data were available for 522/574 (90.9%) participants in the intervention arm and for 484/564 (85.8%) participants in the control arm. For the change in HbA1c, crude analysis data were available for 87/574 (15.2%) participants in the intervention arm and 73/564 (12.9%) participants in the control arm. For the glycaemic control, crude analysis data were available for 89/574 (15.5%) participants in the intervention arm and 79/564 (14.0%) participants in the control arm. For the treatment adherence outcome data were available for 574/574 (100.0%) in the intervention arm and 564/564 (100.0%) in the control arm. For the smoking cessation outcome, data were available for 20/574 (3.5%) in the intervention arm and 23/564 (4.1%) in the control arm.

For the adjusted analyses for the primary outcome, change in DBP and serum cholesterol outcomes data were available for 196/574 (34.1%) participants in the intervention arm and 178/564 (31.6%) participants in the control arm. For the adjusted analysis of the hypertension control outcome, data were available for 152/574 (26.5%) in the intervention arm and 63/564 (11.2%) in the control arm. For the change in HbA1c outcome, data were available for 87/574 (15.2%) in the intervention arm and 73/564 (12.9%) in the control arm. For the glycaemic control outcome, data were available for 27/574 (4.7%) in the intervention arm and 27/564 (4.8%) in the control arm. For the treatment adherence outcome, data were available for 150/574 (26.1%) in the intervention arm and 41/564 (7.3%) in the control arm. For the smoking cessation outcome, 10/574 (1.7%) in the intervention arm and 10/564 (1.8%) in the control arm. The crude intracluster correlation coefficient for the primary outcome was 0.11.

**Table 1.** Baseline characteristics

Characteristics	Intervention, *n* (%)	Control, *n* (%)
**Clusters**		
Total	13 (50.0)	13 (50.0)
**Doctors**		
Male	13 (100.0)	13 (100.0)
Female	0 (0.0)	0 (0.0)
**Paramedics**		
Male	13 (100.0)	13 (100.0)
Female	0 (0.0)	0 (0.0)
**Participants**		
Total	574 (50.4)	564 (49.6)
Mean cluster size (SD)	44.15 (33.22)	43.38 (27.96)
**Sex**		
Male	290 (50.5)	268 (47.5)
Female	284 (49.5)	296 (52.5)
Mean age, years (SD)	45.69 (11.72)	44.60 (12.40)
Mean education, years (SD)	6.5 (4.8)	6.1 (4.4)
Mean BMI, kg/m^2^ (SD)	27.52 (5.85)	26.49 (5.67)
Hypertensive	574 (100)	564 (100)
Smoker	71 (12.4)	90 (16.0)
Mean fasting blood sugar, mg/dL (SD)	134.69 (56.06)	141.88 (55.81)
Mean random blood sugar, mg/dL (SD)	151.99 (70.73)	140.78 (62.43)
Mean HbA1c (%)	8.28 (2.51)	8.026 (2.58)
Mean systolic blood pressure, mmHg (SD)	161.25 (13.28)	161.42 (16.38)
Mean diastolic blood pressure, mmHg (SD)	103.60 (8.91)	103.48 (9.02)
Mean serum cholesterol, mg/dL (SD)	195.06 (45.38)	185.95 (44.03)

Hypertensive defined as baseline SBP >140 mmHg.

A statistically significant effect of the intervention on the primary outcome was found, with a baseline to follow-up reduction in SBP observed in the intervention arm that was statistically significantly greater than the baseline to follow-up reduction in SBP observed in the control arm (Table 2). However, although the intervention produced a clinically significant effect, there was considerable uncertainty about the exact size of this effect owing to the relatively wide CIs around the estimate. For the secondary outcomes, the baseline to follow-up reduction in DBP observed in the intervention arm was statistically significantly greater than the baseline to follow-up reduction in DBP observed in the control arm; however, again, while potentially clinically important, the effect was imprecisely estimated (Table 2). A statistically and clinically significant increase in the proportion of patients with controlled hypertension in the intervention arm versus the control arm was also found, and the baseline to follow-up reduction in total serum cholesterol observed in the intervention arm was statistically significantly greater than the baseline to follow-up increase in total serum cholesterol observed in the control arm (a clinically important effect), as shown in Table 2. However, there was no statistically significant intervention effect on the change in HbA1c or glycaemic control (Table 2).

**Table 2.** Primary and secondary outcomes

	Intervention, mean outcome (95% CI) (clusters *n* = 13)	Control, mean outcome (95% CI) (clusters *n* = 13)	Crude control-intervention difference (95% CI); *P* value^b^	Adjusted control-intervention difference (95% CI); *P* value^b^
**Change in SBP, mmHg^c^**	-25.23 (-29.86 to -20.61)	-9.41 (-21.24 to 2.24)	15.82 (3.60 to 28.04); 0.01	12.63 (0.68 to 24.57); 0.04
**Change in DBP, mmHg^c^**	-18.18 (-22.12 to -14.25)	-8.62 (-15.00 to -2.24)	9.57 (2.39 to 16.74); 0.01	7.58 (0.61 to 14.55); 0.04
**Hypertension control, %^d^**	69.56 (57.09 to 82.03)	35.79 (15.44 to 56.13)	-34pp (-56 to -11); 0.01	-30pp (-53 to -6); 0.02
**Change in HbA1c, %^c^**	-0.70pp (-2.02 to 0.61)	-1.71pp (-2.63 to -0.79)	1.01pp (-2.53 to 0.52); 0.18	0.59pp (-0.29 to 0.17); 0.60
**Glycaemic control, %^e^**	33.03 (10.11 to 55.94%)	42.79 (27.22 to 58.36%)	10pp (-17 to 36); 0.45	3pp (-2 to 87); 0.19
**Change in total serum cholesterol (mg/dL)^c^**	-27.69 (-31.69 to -23.69)	0.52 (-9.85 to 10.89)	28.21 (17.37 to 39.06); 0.001	22.52 (15.89 to 29.16); 0.001
**Treatment adherence^f^**	73.11 (56.34 to 89.88)	18.60 (6.63 to 30.57)	-54.51 (-74.13 to -34.88); 0. 00	-53.57 (-77.68 to -29.46); 0.00
**Smoking cessation**	3.08 (0.78 to 5.38)	4.17 (1.74 to 6.62)	1.10 (-2.08 to 4.28); 0.48	0.03 (-1.37 to 1.43); 0.97

DBP = diastolic blood pressure. pp = percentage points. SBP = systolic blood pressure. ^a^Arm-specific mean outcomes and their 95% confidence intervals are themselves based on cluster-level summary (mean/proportion) outcomes. ^b^All control minus intervention differences (that is, intervention effect estimates) are based on analysis of crude/covariate-adjusted cluster-level summary (mean/proportion) outcomes. ^c^All change outcomes are calculated as outcome at endpoint minus outcome at baseline. ^d^Hypertension control defined as endpoint systolic BP ≤140 mmHg; ^e^Glycaemic control defined as endpoint HbA1c ≤7%. ^f^Treatment adherence was defined as patient having attended ≥5 treatment visits. All differences for percentage outcomes are on the absolute scale as percentage points. All analyses use only complete cases.

Patients were categorised as adherent if they had attended ≥5 visits. Treatment adherence was found to be statistically significantly higher in the intervention arm compared with the control arm. At the final visit (that is, the 9th follow-up) all the patients were traced. The loss to follow-up rates differed between two arms with 9% in the intervention arm and 14% in the control arm. However, no statistically significant difference was found in the smoking cessation between both the arms. A total of 57 patients (intervention *n* = 17; control *n* = 40) were hospitalised for ≥1 days for a medical condition during the 9-month treatment. There were not enough reported events leading to hospitalisation for a meaningful statistical analysis, but they included: 21 cardiovascular (intervention* n* = five; control *n* = 16), seven cerebrovascular (intervention: one; control: six); and 29 undefined diagnosis (intervention *n* = 11; control *n* = 18).

No evidence for any modification of the intervention’s effect on the primary outcome owing to sex was found (Table 3).

**Table 3.** Effect modification by sex for the primary outcome (change in SBP)

	Mean outcome (95% CI)^a^			
	Intervention (clusters *n* = 13)	Control (clusters *n* = 13)	Crude effect (95% CI); *P* value^b^	Adjusted effect (95% CI); *P* value
**Female change in SBP**	-25.62 (-30.49 to -20.75)	-10.42 (-21.92 to 1.09)	15.20 (3.06 to 27.34); 0.02	13.32 (0.74 to 25.89); 0.04
**Male change in SBP**	-24.64 (-29.33 to -19.95)	-7.52 (-23.42 to 8.39)	17.13 (0.81 to 33.44); 0.04	17.51 (3.69 to 31.34); 0.02
**Female–male** **difference** **for change in SBP**	-	-	-1.93 (-12.38 to 8.53); 0.70	-0.50 (-9.27 to 8.27); 0.90

SBP = systolic blood pressure. ^a^Arm-specific mean outcomes and their 95% confidence intervals are themselves based on cluster-level summary mean outcomes for females/males/female–male differences. ^b^All control minus intervention differences (intervention effect estimates) are based on analysis of crude/covariate-adjusted cluster-level summary (mean/proportion) female–male difference outcomes. Change in SBP is calculated as outcome at endpoint minus outcome at baseline.

## Discussion

### Summary

In Pakistan, this is the first pragmatic cluster randomised trial of engaging private healthcare providers to deliver hypertension–CVD risk reduction intervention. Importantly, a provincial NCD control programme-endorsed hypertension–CVD care package has been integrated into general practice clinics; that is, outside the specialist domain.

The enhanced package, with standardised prescription, adherence support, and lifestyle modifications, was offered in the intervention clusters of private clinics, and was found more effective in terms of achieving BP and cholesterol control at 9 months of completed treatment. In this trial, the better control of BP and cholesterol in the intervention cluster was potentially a combined effect of the care components. It is not possible to separate the individual effects of standardised prescription, adherence support, and lifestyle modification.

### Strengths and limitations

The strengths of the study are that: (a) private clinics in the trial were already engaged in communicable disease (TB) control and, therefore, had prior experience of working with research partners and respective district health offices; (b) the care and research protocols were designed and implemented with a view to enhancing the replicability and sustainability.^27^

A major limitation of the study was that neither healthcare providers nor patients were blinded to intervention or control clusters. To minimise the risk of enrolment difference in two arms (if different diagnostic criteria and/or equipment had been allowed) the diagnosis and record-keeping in the control clusters was reinforced as in the intervention arm. This is likely to have reduced the measured effect between the intervention and control arms, because the influence of better diagnosis and record-keeping components of the intervention was the same in both arms. Another limitation is the relatively short (9 month) follow-up time. These limitations may have negated potential differences in measured outcomes between the intervention and control clusters. However, collecting patient outcomes at 9 months was felt to be necessary to cope with the time and resource constraints.

### Comparison with existing literature

The assumed additional contribution of the lifestyle modification and adherence support to the better control of BP and cholesterol corroborates with studies in India and Bangladesh. A randomised controlled trial conducted in India that assessed the influence of comprehensive lifestyle interventions at 3 and 6 month follow-up, showed significant improvements in hypertension control, lipid profile disorders, and obesity.^28^ Similarly, in Bangladesh, offering health advice to newly diagnosed patients with hypertension after 4–6 months of follow-up showed that half of all patients achieved their BP control target (≤140/90 mmHg).^29^

Adherence to monthly follow-up visits was significantly better in the intervention clusters compared with the control. The attribution of better patient adherence to active follow-up procedures and telephone reminders in the intervention clusters corroborates findings of a study in Cameroon, where patient retention for diabetes and hypertension management in rural districts was enhanced by regular contact between healthcare providers and patients.^30^

These findings are important as the private sector is considered a major source of primary health care in urban localities of Pakistan.^16^ Furthermore, the district-steered partnership, in which the respective district health offices enable and support the private clinics, is considered replicable and sustainable within the routine district healthcare system and with minimal additional resource implications.

### Implications for research and practice

A process evaluation of the trial, published in this journal,^20^ indicated that offering programme-endorsed hypertension care at private clinics, during the first 9 months after registration, was feasible for the clinic staff and acceptable to the patients.

Long-term care of hypertension, hyperlipidaemia, and other CVD risk factors at private urban clinics is an important healthcare consideration. The private clinics are generally accustomed to offering relatively short-term care of curable conditions. Therefore, assessment of effectiveness and feasibility of private clinics offering long-term care (longer than the 9 months follow-up of this study) of hypertension, requires further prospective studies with longer follow-up periods, such as 5 or 10 years.

This pragmatic randomised controlled trial in private clinics in underserved urban areas has shown that implementation of an enhanced care package of newly diagnosed hypertension, and its associated conditions, compared with enhanced diagnosis and data recording processes alone, was associated with better control of SBP, DBP, and serum cholesterol levels. The findings of the trial, supplemented by the process evaluation study, support the scaling of this intervention for better care of hypertension and associated conditions in urban population of Punjab and other provinces of Pakistan.

## Trial registration

The trial was registered with the Current Controlled Trials (reference number: ISRCTN34381594).
